# Posttraumatic stress disorder with psychotic symptoms. A case report

**DOI:** 10.1192/j.eurpsy.2021.1996

**Published:** 2021-08-13

**Authors:** R. Galeron, M. Jiménez Cabañas, P. Albarracin, E. Herrero, M. Huete Naval, B. Serván Rendón-Luna

**Affiliations:** 1 Psychiatry And Mental Health, Hospital Clínico San Carlos, Madrid, Spain; 2 Psychiatry And Mental Health, Hospital Clinico San Carlos, Madrid, Spain

**Keywords:** psychosis, Posttraumatic Stress Disorder, trauma

## Abstract

**Introduction:**

We present a 29-year-old man with a family psychopathological history of depression and a personal history of Posttraumatic Stress Disorder after sexual and psychological abuse in childhood, depressive symptoms and substance use (cannabis), who experienced delusions that made him feel threatened and in danger, with huge anxiety and insomnia for one year after a heartbreak. In addition, the patient was dysphoric, verborrheic and presented ruminative thoughts and flashbacks of abuse suffered in childhood.

**Objectives:**

To review the literature of Posttraumatic Stress Disorder with Psychotic Symptoms (PTSD-PS) and study the difference between PTSD-PS and other psychotic disorders.

**Methods:**

Literature review of scientific articles searching in Pubmed and Medline. We considered articles in English and Spanish.

**Results:**

Pharmacological treatment with antipsychotics and mood stabilizer was started with remission of anxiety and insomnia and recovery of euthymia. Delusions persisted but without affective and behavioral repercussions. With psychotherapeutic work in a psychiatric Day Hospital, complete remission and proper processing of traumatic experiences were achieved. The main psychotic symptoms in PTSD are hallucinations and delusions which tend to chronicity. The content is often paranoid and persecutory in nature but not complex or bizarre like those found in schizophrenia. These symptoms are not limited to flashback episodes and the content may or may not be trauma related.

**Conclusions:**

Although the studies show PTSD-PS presents characteristic symptoms, more research about is needed.

**Disclosure:**

No significant relationships.

